# Improved Electrochemical Properties of LiMn_2_O_4_-Based Cathode Material Co-Modified by Mg-Doping and Octahedral Morphology

**DOI:** 10.3390/ma12172807

**Published:** 2019-08-31

**Authors:** Hongyuan Zhao, Yongfang Nie, Dongyang Que, Youzuo Hu, Yongfeng Li

**Affiliations:** 1 School of Mechanical and Electrical Engineering, Henan Institute of Science and Technology, Xinxiang 453003, China; 2 Zhumadian Power Supply Company, State Grid Henan Electric Power Company, Zhumadian 463500, China; 3 Chemistry Department, Lancaster University, Lancaster LA1 4YB, UK

**Keywords:** LiMn_2_O_4_, Mg-doping, octahedral morphology, synergistic effect, electrochemical properties

## Abstract

In this work, the spinel LiMn_2_O_4_ cathode material was prepared by high-temperature solid-phase method and further optimized by co-modification strategy based on the Mg-doping and octahedral morphology. The octahedral LiMn_1.95_Mg_0.05_O_4_ sample belongs to the spinel cubic structure with the space group of Fd3m, and no other impurities are presented in the XRD patterns. The octahedral LiMn_1.95_Mg_0.05_O_4_ particles show narrow size distribution with regular morphology. When used as cathode material, the obtained LiMn_1.95_Mg_0.05_O_4_ octahedra shows excellent electrochemical properties. This material can exhibit high capacity retention of 96.8% with 100th discharge capacity of 111.6 mAh g^−1^ at 1.0 C. Moreover, the rate performance and high-temperature cycling stability of LiMn_2_O_4_ are effectively improved by the co-modification strategy based on Mg-doping and octahedral morphology. These results are mostly given to the fact that the addition of magnesium ions can suppress the Jahn–Teller effect and the octahedral morphology contributes to the Mn dissolution, which can improve the structural stability of LiMn_2_O_4_.

## 1. Introduction

With the increasingly serious environmental pollution, new energy and environmental technology have caught more and more extensive attention. Under this circumstance, the research and development of lithium-ion batteries are receiving more and more attention at home and abroad since their first commercial application in 1991 [1,2,3]. As an important cathode material, LiMn_2_O_4_ possesses a rather high cost advantage because of the abundant manganese resource and this material can be obtained by many preparation technologies [4,5,6,7,8]. Moreover, this material does not involve the use of toxic metal elements. All these advantages can promote large-scale applications of LiMn_2_O_4_. It must be noted, however, that the cycling stability and high temperature performance cannot meet the requirement of long endurance mileage [9,10,11,12].

According to the existing literature, the electrochemical performance of LiMn_2_O_4_ can be severely affected by the Jahn–Teller distortion effect and Mn dissolution during the process of discharging and charging due to the fact that the Jahn–Teller distortion and Mn dissolution is closely related to the trivalent state (Mn^3+^), which can seriously affect the discharging process and the discharged state [13,14,15,16]. In recent years, many optimization strategies (doping, coating, morphology control, etc.) have been developed to address these problems [11,15,17,18,19,20,21]. Among them, the doping strategy usually choses other heterogeneous ions (Li^+^, Mg^2+^, Zn^2+^, Al^3+^, Cr^3+^, Si^4+^, etc.) to replace a small amount of manganese ions [2,9,22,23,24,25]. As a result, the Jahn–Teller distortion effect can be decreased, which enhances the structural stability of LiMn_2_O_4_. Among these heterogeneous ions, magnesium has a wide distribution in nature and can work as an additive in electrolyte as well as an additive in cathode slurry [26,27]. More importantly, the addition of magnesium ions in LiMn_2_O_4_ can play a positive role in improving the electrochemical properties. Huang et al. [23] have prepared Mg-doped LiMn_2_O_4_ samples and investigated the effect of introducing magnesium ions on the structure, morphology, and cycling properties. The introduction of magnesium ions can strengthen the structural stability of LiMn_2_O_4_ by reducing the cell volume, and the reduction of trivalent manganese ions further strengthens the crystal structure of LiMn_2_O_4_ by suppressing the Jahn–Teller effect. The obtained Mg-doped LiMn_2_O_4_ sample can show higher capacity retention. Many other research works have confirmed the positive effect of introducing magnesium ions on optimizing the cycling properties of LiMn_2_O_4_ [28]. Furthermore, it has been reported that the high-performance LiMn_2_O_4_ can be prepared by solid-state method using Mn_3_O_4_ with octahedral morphology [29]. Zhao et al. [30] successfully prepared the octahedral LiMn_2_O_4_ particles by using Mn_3_O_4_ octahedra as manganese source. Since the octahedral morphology can suppress the dissolution of Mn to steady crystal structure, the obtained LiMn_2_O_4_ octahedra shows excellent electrochemical properties. Based on the above analysis, it is worth considering that the simultaneous use of the Mg-doping and octahedral morphology may greatly enhance the electrochemical properties of LiMn_2_O_4_.

Herein, the Mg-doped LiMn_2_O_4_ octahedra were prepared by high temperature solid-phase method with magnesium nitrate and Mn_3_O_4_ octahedra as doping agent and manganese source. The electrochemical properties of the octahedral LiMn_1.95_Mg_0.05_O_4_ sample as cathode material were investigated in detail. It could be found that the electrochemical properties of LiMn_2_O_4_ were greatly enhanced by jointly using the Mg-doping and octahedral morphology. This work indicates that the co-modification strategy based on Mg-doping and octahedral morphology has vital significance to promote the practical application of LiMn_2_O_4_.

## 2. Materials and Methods 

The octahedral LiMn_1.95_Mg_0.05_O_4_ (LMMOO) particles were prepared by high temperature solid-phase method with magnesium nitrate and Mn_3_O_4_ octahedra as doping agent and manganese source. The octahedral Mn_3_O_4_ particles were firstly prepared via a hypothermal approach according to the existing literature [30]. Subsequently, in a typical synthesis process, stoichiometric LiOH·H_2_O, Mn_3_O_4_ octahedra, and Mg(NO_3_)_2_·6H_2_O were ground to obtain the slurry mixture with the help of absolute ethanol. Then, the homogeneous mixture was dried in a drying oven and sintered at 700 °C for 10 h in air. In order to allow the comparison, the undoped LiMn_2_O_4_ (LMO) and LiMn_1.95_Mg_0.05_O_4_ (LMMO) particles were prepared by using electrolytic MnO_2_ as manganese precursor.

The structure and morphology usually have an important impact on the electrochemical properties of cathode material. The obtained LiMn_2_O_4_ and LiMn_1.95_Mg_0.05_O_4_ samples were characterized by using XRD and SEM techniques. The effects of Mg-doping and octahedral morphology on the cycling stability of LiMn_2_O_4_ were studied by fabricating the coin cells with the obtained spinels as cathode materials. The positive electrode was constituted from 85% synthesized product as cathode material, 10% acetylene black as conductive agent, and 5% polyvinylidene fluoride dissolved in N-methyl-2-pyrrolidone as binder. The metallic lithium foil was used as counter electrode, the polypropylene microporous membrane was used as diaphragm, and the 1 M lithium hexafluorophosphate (LiPF_6_) solution in a mixture of the ethylene carbonate (EC) and diethyl carbonate (DEC) at a volume ratio of 1:1 was used as the electrolyte. All the electrochemical tests were carried out on LANHE CT2001A system (LANHE, Wuhan, China) and CHI660E electrochemical workstation (CH Instruments, Shanghai, China).

## 3. Results and Discussion

**Figure 1:**
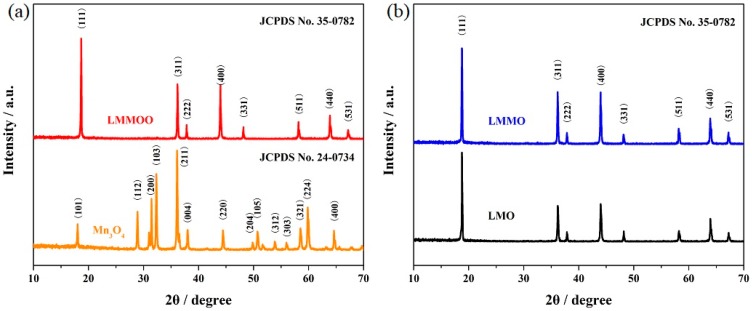
XRD patterns of (**a**) Mn_3_O_4_ and octahedral LiMn_1.95_Mg_0.05_O_4_, and (**b**) LiMn_2_O_4_ and LiMn_1.95_Mg_0.05_O_4_ samples.

In order to confirm the structures of the obtained samples, the LiMn_2_O_4_, LiMn_1.95_Mg_0.05_O_4_, and octahedral LiMn_1.95_Mg_0.05_O_4_ samples were characterized. It can be seen from Figure 1 that the diffraction peaks of the undoped LiMn_2_O_4_ are in good agreement with the standard diffraction peaks of LiMn_2_O_4_ (JCPDS No. 35-0782). No other diffraction peaks of manganese oxide and magnesium oxide can be observed, suggesting the complete transformation of electrolytic manganese dioxide to LiMn_2_O_4_ [31]. After introducing a small amount of magnesium ions, the obtained LiMn_1.95_Mg_0.05_O_4_ sample was still present in the spinel cubic structure of LiMn_2_O_4_, which indicates that the addition of magnesium ions did not change the crystal structure [32,33]. For the LiMn_1.95_Mg_0.05_O_4_ sample obtained from Mn_3_O_4_ octahedra, the characteristic diffraction peaks were indexed to the spinel LiMn_2_O_4_. Moreover, the corresponding peak intensities are stronger than that of the LiMn_2_O_4_ and LiMn_1.95_Mg_0.05_O_4_ samples prepared from electrolytic manganese dioxide, suggesting the good crystalline quality of the octahedral LiMn_1.95_Mg_0.05_O_4_ sample [9,34]. Table 1 lists the related crystal parameters of these three samples. The addition of magnesium ions leads to the reduction of the lattice parameter and shrinking of unit cell volume, suggesting the more stable structural stability of the LiMn_1.95_Mg_0.05_O_4_ samples.

**Figure 2:**
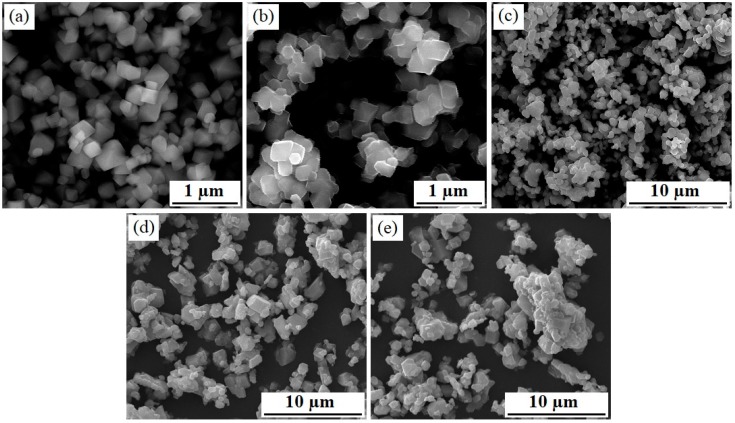
SEM images of (**a**) Mn_3_O_4_ octahedra, (**b**,**c**) LiMn_1.95_Mg_0.05_O_4_ octahedra, (**d**) LiMn_2_O_4_, and (**e**) LiMn_1.95_Mg_0.05_O_4_.

Figure 2 shows the SEM images of the octahedral Mn_3_O_4_, LiMn_2_O_4_, and Mg-doped LiMn_2_O_4_ samples. As shown in Figure 2a, the Mn_3_O_4_ particles prepared by hydrothermal approach present rather good octahedral morphology. For the octahedral LiMn_1.95_Mg_0.05_O_4_ sample shown in Figure 2b,c, it can be seen that it presents a narrow size distribution with regular morphology, which indicates that the LiMn_1.95_Mg_0.05_O_4_ sample inherits the special morphology of Mn_3_O_4_ octahedra [30]. Moreover, the particle size belongs to the submicron scale, which agrees with the particle size of Mn_3_O_4_. By contrast, the particle morphology of the undoped LiMn_2_O_4_ particles (Figure 2d) is irregular with micron grade particle size. Especially, the obvious agglomerated particle can be observed in the undoped LiMn_2_O_4_ particles. These unsatisfactory characteristics usually have a greater negative impact on the electrochemical properties of the cathode material [35,36]. For the LiMn_1.95_Mg_0.05_O_4_ sample (Figure 2e), it shows relatively good size distribution, which is closely related to the addition of a certain amount of magnesium ions, which agrees with the research result [23,33]. These results suggest that the combination of Mg-doping and octahedral morphology can be useful in optimizing the morphology and size distribution of LiMn_2_O_4_ particles.

**Figure 3:**
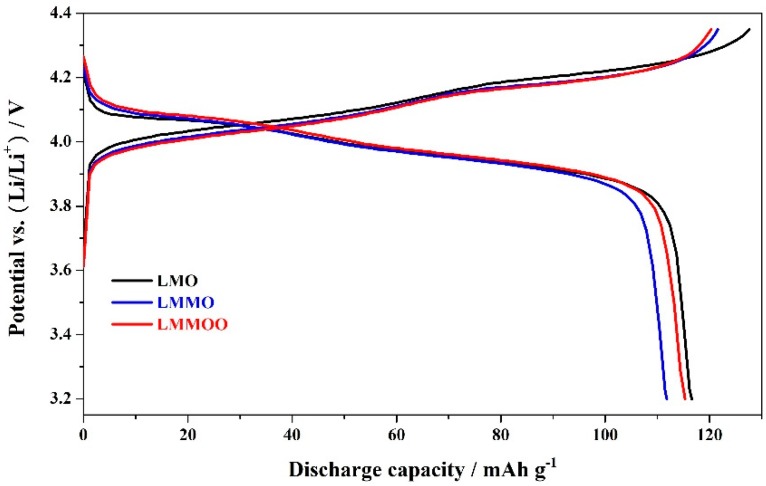
Initial charge–discharge curves of the LiMn_2_O_4_, LiMn_1.95_Mg_0.05_O_4_, and octahedral LiMn_1.95_Mg_0.05_O_4_ samples at 1.0 C.

To investigate the influence of jointly using the Mg-doping and octahedral morphology on the electrochemical performance, the LiMn_2_O_4_, LiMn_1.95_Mg_0.05_O_4_, and octahedral LiMn_1.95_Mg_0.05_O_4_ samples were tested at a cycling rate of 1.0 C, the corresponding initial charge–discharge curves are shown in Figure 3. It can be seen that the undoped LiMn_2_O_4_ sample exhibits characteristic discharge curves of LiMn_2_O_4_ with two voltage plateaus. According to the research result [37,38], these two voltage plateaus correspond to the intercalation/de-intercalation processes of lithium ions, which correspond to the two-phase equilibrium of λ-MnO_2_/Li_0.5_Mn_2_O_4_ and single-phase equilibrium of Li_0.5_Mn_2_O_4_/LiMn_2_O_4_, respectively. For the LiMn_1.95_Mg_0.05_O_4_ and octahedral LiMn_1.95_Mg_0.05_O_4_ samples, the initial charge–discharge curves show similar platform characteristics, and the potential interval of the Mg-doped spinels is less than that of the undoped spinel, suggesting the higher reaction kinetics of the Mg-doped spinels [39]. It is important to note, however, that the discharge voltage plateaus of the LiMn_1.95_Mg_0.05_O_4_ samples are slightly higher than that of the undoped LiMn_2_O_4_ sample, which may be related to the optimization of the Li^+^ intercalation/deintercalation behaviors due to the addition of other cations in the spinel structure [10,33,39,40].

**Figure 4:**
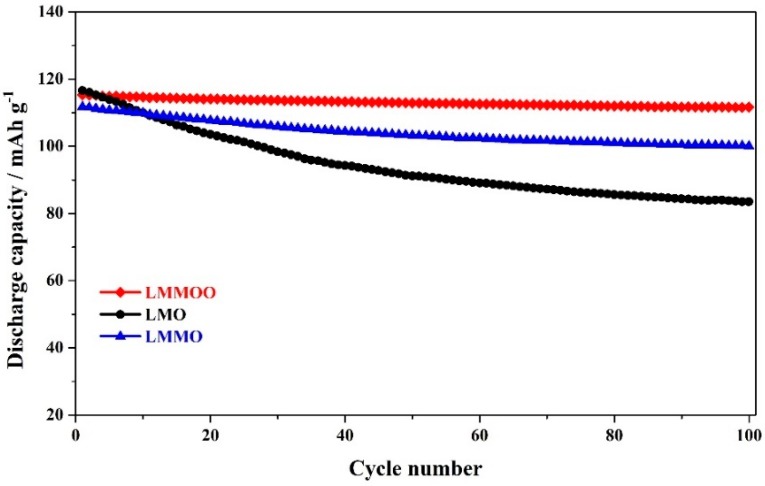
Cycling stability of the LiMn_2_O_4_, LiMn_1.95_Mg_0.05_O_4_, and octahedral LiMn_1.95_Mg_0.05_O_4_ samples at 1.0 C.

The cycling performance is a very important index sign for the practical application of LiMn_2_O_4_. Figure 4 shows the cycling stability of the LiMn_2_O_4_, LiMn_1.95_Mg_0.05_O_4_, and octahedral LiMn_1.95_Mg_0.05_O_4_ samples at 1.0 C. For the undoped LiMn_2_O_4_ sample, it exhibits an initial capacity of 116.6 mAh g^−1^ with unsatisfactory cycling stability. After 100 cycles, the discharge capacity presents much decrease with the 100th capacity of 83.5 mAh g^−1^. Such poor performance is mainly attributed to the wide size distribution and large agglomerated particle [35]. When adding some magnesium ions, the LiMn_1.95_Mg_0.05_O_4_ sample shows higher capacity retention than that of undoped LiMn_2_O_4_ sample. Although the addition of magnesium ions decreases the initial discharge capacity, the capacity retention of the LiMn_1.95_Mg_0.05_O_4_ sample is enhanced greatly. After 100 cycles, the discharge capacity can maintain 100.1 mAh g^−1^ with high retention of 89.5%. The improvement in cycling stability is attributed to the fact that the introduction of magnesium ions can strengthen the structural stability of LiMn_2_O_4_ by inhibiting the Jahn–Teller effect and reducing the cell volume [23,33]. It is important to note that the octahedral LiMn_1.95_Mg_0.05_O_4_ sample can show more excellent cycling performance. Compared with the undoped LiMn_2_O_4_ and LiMn_1.95_Mg_0.05_O_4_ samples, the capacity retention of the octahedral LiMn_1.95_Mg_0.05_O_4_ sample can reach up to 96.8% after 100 cycles with the 100th capacity of 111.6 mAh g^−1^. Such excellent cycling stability mainly benefits from the synergistic effect of the Mg-doping and octahedral morphology. The Mg-doping can suppress the Jahn–Teller effect and the octahedral morphology can contribute to inhibit the Mn dissolution, which can improve the structural stability of LiMn_2_O_4_ [29,36].

**Figure 5:**
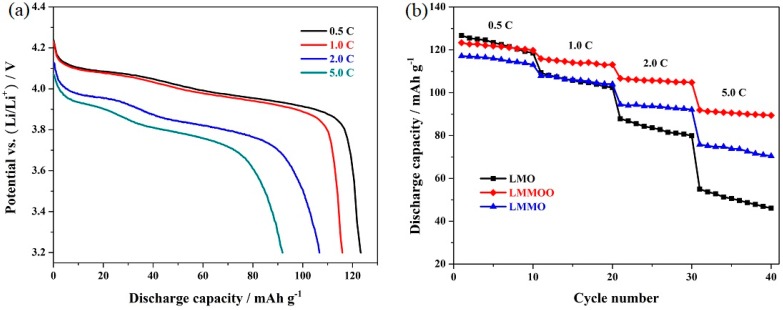
(**a**) Representative discharge curves of the octahedral LiMn_1.95_Mg_0.05_O_4_ sample and (**b**) rate capability of the LiMn_2_O_4_, LiMn_1.95_Mg_0.05_O_4_, and octahedral LiMn_1.95_Mg_0.05_O_4_ samples.

**Figure 6:**
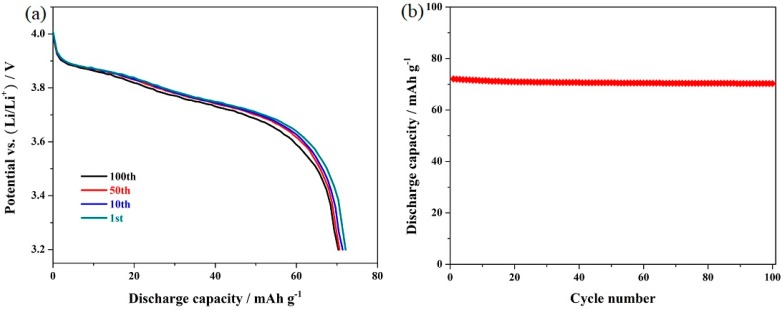
(**a**) Initial discharge curves and (**b**) cycling performance of the octahedral LiMn_1.95_Mg_0.05_O_4_ sample at 10 C.

To investigate the effect of jointly using the Mg-doping and octahedral morphology on the rate capability, the LiMn_2_O_4_, LiMn_1.95_Mg_0.05_O_4_, and octahedral LiMn_1.95_Mg_0.05_O_4_ samples were successively cycled at 0.5, 1.0, 2.0, and 5.0 C, respectively. Figure 5a presents the characteristic discharge curves of the octahedral LiMn_1.95_Mg_0.05_O_4_ sample (the representative of these three samples) at different cycling rates. As shown here, the discharge capacity and voltage platform are significantly affected by the high cycling rate. When the cycling rate gradually increases, the boundary of the two voltage plateaus become smooth and fuzzy and the discharge capacity gradually decreases due to the increased polarization, which are in accordance with the existing literature [41,42,43]. Figure 5b shows the corresponding cycling performance of the LiMn_2_O_4_, LiMn_1.95_Mg_0.05_O_4_, and octahedral LiMn_1.95_Mg_0.05_O_4_ samples at varying cycling rates. The undoped LiMn_2_O_4_ sample exhibits a discharge capacity of 126.7 mAh g^−1^ at low cycling rate of 0.5 C. With the increase of the cycling rates, the discharge capacity is influenced greatly. As the cycling rate increases to 5.0 C, the undoped LiMn_2_O_4_ sample only exhibits 55.0 mAh g^−1^ with rather low retention of 43.4%. By contrast, the Mg-doped LiMn_2_O_4_ samples present outstanding cycling stability at high cycling rate. Especially, the octahedral LiMn_1.95_Mg_0.05_O_4_ sample can exhibit a higher capacity of 91.8 mAh g^−1^ at a high cycling rate of 5.0 C. To further explore the high-rate cycling stability, the octahedral LiMn_1.95_Mg_0.05_O_4_ samples were cycled at 10 C. The corresponding characteristic discharge curves are shown in Figure 6a. It can be found that the characteristic voltage plateaus in the discharge curves become blurred to a large extent, which agrees with the research result [39,44,45]. Figure 6b presents the cycling performance of the octahedral LiMn_1.95_Mg_0.05_O_4_ samples at 10 C. It can show satisfactory retention of 97.5% after 100 cycles with initial capacity of 72.1 mAh g^−1^. The above results suggest that the co-modification strategy based on Mg-doping and octahedral morphology is an important means for effectively improving the rate capability of LiMn_2_O_4_.

**Figure 7:**
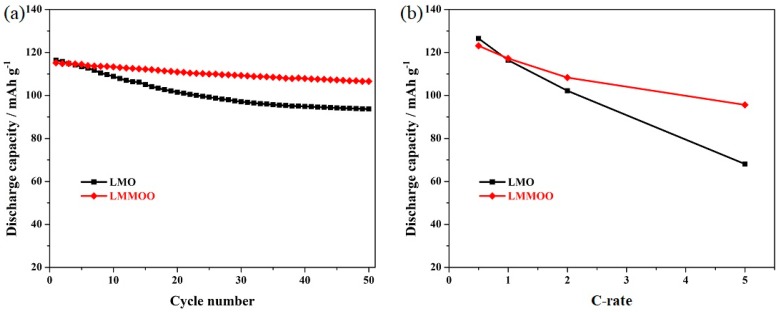
(**a**) Cycling stability of the LiMn_2_O_4_ and octahedral LiMn_1.95_Mg_0.05_O_4_ samples at 1.0 C under high temperature (55 °C), and (**b**) representative discharge curves of the octahedral LiMn_1.95_Mg_0.05_O_4_ sample.

Figure 7a presents the high-temperature cycling stability of the LiMn_2_O_4_ and octahedral LiMn_1.95_Mg_0.05_O_4_ samples at 1.0 C. It can be seen that the cycling stability of the undoped LiMn_2_O_4_ sample is much poorer than that of the octahedral Mg-doped LiMn_2_O_4_ sample. The initial discharge capacity of the undoped LiMn_2_O_4_ sample is comparable to the test results shown in Figure 4, but the capacity retention is rather poor. After 50 cycles, this sample only presents low capacity retention of 80.5%. It is important to note that the octahedral LiMn_1.95_Mg_0.05_O_4_ can present excellent capacity retention of 92.5% with a satisfactory 50th discharge capacity of 106.6 mAh g^−1^. Figure 7b shows the rate capability of the LiMn_2_O_4_ and octahedral LiMn_1.95_Mg_0.05_O_4_ samples at 55 °C. As shown here, the octahedral LiMn_1.95_Mg_0.05_O_4_ sample shows more stable high-temperature cycling stability at different rates, especially the high cycling rate. When tested at 5.0 C, the octahedral LiMn_1.95_Mg_0.05_O_4_ sample can maintain the discharge capacity of 95.6 mAh g^−1^. Unfortunately, the undoped LiMn_2_O_4_ sample presents unsatisfactory rate capability, which further confirms the synergistic effect of the Mg-doping and octahedral morphology.

**Figure 8:**
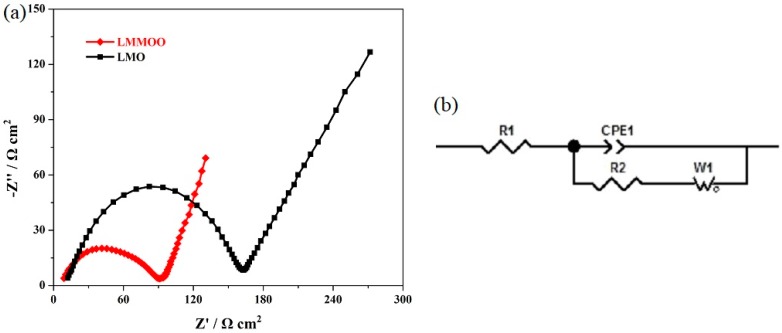
(**a**) Nyquist plots of the LiMn_2_O_4_ and octahedral LiMn_1.95_Mg_0.05_O_4_ samples and (**b**) equivalent circuit model of EIS.

Figure 8a presents the Nyquist plots of the LiMn_2_O_4_ and octahedral LiMn_1.95_Mg_0.05_O_4_ samples, and Figure 8b shows the corresponding equivalent circuit model. According to the research result [9,10,23], the charge transfer resistance (R_2_) in the high-frequency region has strong ties to the electrochemical properties. Therefore, we mainly studied the R_2_ value to confirm the effect of both the Mg-doping and octahedral morphology on the electrochemical performance. As shown in Figure 8a, the combination of Mg-doping and octahedral morphology produces an important influence on the R_2_ value. The addition of magnesium ions can suppress the Jahn–Teller distortion effect to improve the structural stability, and the octahedral morphology of LiMn_2_O_4_ octahedra suppresses the dissolution of Mn in electrolyte [30,36]. Moreover, the uniform particle size distribution also contributes to the diffusion efficiency of lithium ions [33,39,43]. As a result, the octahedral LiMn_1.95_Mg_0.05_O_4_ sample presents lower initial charge-transfer resistance than that of the undoped spinel, which suggests excellent electrochemical properties. 

## 4. Conclusions

To summarize, the octahedral LiMn_1.95_Mg_0.05_O_4_ sample was prepared by high temperature solid-phase method with magnesium nitrate and Mn_3_O_4_ octahedra as the doping agent and manganese source. XRD and SEM results indicate that the Mg-doping does not change the structure of LiMn_2_O_4_ and the octahedral morphology of manganese source is inherited well in the obtained LiMn_1.95_Mg_0.05_O_4_ sample. The synergistic effect of both the Mg-doping and octahedral morphology on the electrochemical performance were confirmed. The octahedral LiMn_1.95_Mg_0.05_O_4_ sample can show more excellent electrochemical properties compared to the undoped LiMn_2_O_4_ and LiMn_1.95_Mg_0.05_O_4_ particles. When cycled at 1.0 C, the capacity retention of the LiMn_1.95_Mg_0.05_O_4_ sample can reach up to 96.8% after 100 cycles with the initial capacity of 115.3 mAh g^−1^. Not only that, the combination of Mg-doping and octahedral morphology also significantly enhances the rate capability and high-temperature performance. This work is meaningful to promote the large-scale commercial application of LiMn_2_O_4_.

## Figures and Tables

**Table 1:** Crystal parameters of the LiMn_2_O_4_ and LiMn_1.95_Mg_0.05_O_4_ samples.

Sample	Space	a (nm)	V (nm^3^)
LMO	Fd-3m	0.82392	0.55931
LMMO	Fd-3m	0.82287	0.55718
LMMOO	Fd-3m	0.82253	0.55649
